# Breast milk microRNAs: Potential players in oral tolerance development

**DOI:** 10.3389/fimmu.2023.1154211

**Published:** 2023-03-14

**Authors:** Emelie Ahlberg, Ahmed Al-Kaabawi, Rebecka Thune, Melanie Rae Simpson, Sindre Andre Pedersen, Erika Cione, Maria Christina Jenmalm, Lina Tingö

**Affiliations:** ^1^ Division of Inflammation and Infection, Department of Biomedical and Clinical Sciences, Linköping University, Linköping, Sweden; ^2^ Department of Public Health and Nursing, Norwegian University of Science and Technology (NTNU), Trondheim, Norway; ^3^ Library Section for Research Support, Data and Analysis, NTNU University Library, Norwegian University of Science and Technology (NTNU), Trondheim, Norway; ^4^ Department of Pharmacy, Health and Nutritional Sciences, University of Calabria, Rende, Cosenza, Italy; ^5^ Nutrition-Gut-Brain Interactions Research Centre, School of Medical Sciences, Örebro University, Örebro, Sweden; ^6^ Food and Health Programme, Örebro University, Örebro, Sweden

**Keywords:** micro-RNA, non-coding RNA, dendritic cells, oral tolerance, regulatory T cell, allergy, infant - age, breast milk

## Abstract

Breast milk is an essential source of nutrition and hydration for the infant. In addition, this highly complex biological fluid contains numerous immunologically active factors such as microorganisms, immunoglobulins, cytokines and microRNAs (miRNAs). Here, we set out to predict the function of the top 10 expressed miRNAs in human breast milk, focusing on their relevance in oral tolerance development and allergy prevention in the infant. The top expressed miRNAs in human breast milk were identified on basis of previous peer-reviewed studies gathered from a recent systematic review and an updated literature search. The miRNAs with the highest expression levels in each study were used to identify the 10 most common miRNAs or miRNA families across studies and these were selected for subsequent target prediction. The predictions were performed using TargetScan in combination with the Database for Annotation, Visualization and Integrated Discovery. The ten top expressed miRNAs were: let-7-5p family, miR-148a-3p, miR-30-5p family, miR-200a-3p + miR-141-3p, miR-22-3p, miR-181-5p family, miR-146b-5p, miR-378a-3p, miR-29-3p family, miR-200b/c-3p and miR-429-3p. The target prediction identified 3,588 potential target genes and 127 Kyoto Encyclopedia of Genes and Genomes pathways; several connected to the immune system, including TGF-b and T cell receptor signaling and T-helper cell differentiation. This review highlights the role of breast milk miRNAs and their potential contribution to infant immune maturation. Indeed, breast milk miRNAs seem to be involved in several pathways that influence oral tolerance development.

## 1 Introduction

Breast milk is an important source of nutrition and hydration for infant mammals, including humans (1). However, this highly complex biological fluid has functions that exceed nutrition (2, 3). Breast milk contains a number of immunological factors such as microorganisms, immunoglobulins and cytokines that represent the first postnatal immunological stimuli and protection for the infant (3, 4). The composition of breast milk changes over time to the different needs of the infant. For example, the first milk produced, colostrum, contains a higher concentration of immunoglobulin A compared to mature milk (5). Not only does the milk offer protective effects against infections in early life, but it may also have more long-lasting immune modulatory effects (6–9).

Amongst the immune regulatory components in breast milk, microRNA (miRNA) represents an abundant form. The miRNAs are small, non-coding fragments of RNA, with the potential to regulate gene expression by post-transcriptional modifications of mRNA strands (10). During the last 15 years, miRNAs have gained increasing interest as research has moved forward through new technologies and discoveries; indeed, it has been suggested that miRNAs are able to regulate up to 60% of all transcribed mRNAs (11, 12). Although present in all biological fluids, miRNAs are most abundant in breast milk and, interestingly, many of the milk-born miRNAs seem to be evolutionarily conserved between different mammal species (13–15). Although the biological relevance remains to be clarified, this conservation of sequence suggest that miRNAs serve fundamental functions. Like other breast milk components, the miRNA profile seems to vary over time, displaying daily as well as more long-term fluctuations (14, 16, 17), although empirical data is still scarce. An astonishing >1,900 different types of miRNAs have been detected in human breast milk, with potential biological implications on cell communication, fatty acid biosynthesis, regulation of actin skeleton and a vast number of immunological pathways (12).

Human milk miRNAs hypothetically regulate infant gene expression through epigenetic modifications and thereby affect various biological processes, including immune maturation. These epigenetic effects could potentially occur both up-stream and down-stream of gene transcription. Down-stream of transcription, a miRNA could target one or more mRNA and inhibit their translation into proteins. For example, miRNA-155, expressed in human milk (14), targets the signal transducer and activator of transcription (STAT) 1 mRNA and thereby, *via* concurrent activation of IL-2/STAT5 signaling, promote regulatory T cell (Treg) differentiation (18, 19). Up-stream of gene transcription, miRNAs could for example influence gene expression *via* DNA methylation, interfering with the transcriptional process. For example, DNA de/methylation regulates FOXP3 expression, a key transcription factor in the differentiation of CD4+ T cells into Tregs (20, 21). DNA methyltransferase (DNMT) 1 and DNMT3b seem to be in control of this transcriptional “switch” (22). Interestingly, one of the most highly expressed miRNAs in mammalian milk, miRNA-148a, can downregulate the expression of DNA methyltransferase 1 *in vitro* (23). Also, Admyre et al. (24) observed how human milk EVs could dose-dependently increase the numbers of FOXP3+CD4+CD25+ Treg cells in peripheral blood mononuclear cells. However, it remains to be further explored if such effects can be exclusively attributed to the EV born miRNAs. On this note, it should also be pointed out that although interactions between different bioactive components in the milk likely occurs – for example miR-155 and miR-181 interacts with TGF-β and IL-10 (all abundantly found in breast milk) to regulate proliferation and function of Tregs (18) - functional studies designed to uncover details in these interplays are missing. Yet, early discoveries, as highlighted here, prompts enthralling questions about the biological relevance of human breast milk miRNAs and their role in infant development and immune maturation.

### 1.1 Transfer of miRNAs from mother to child

Breast milk, along with all biological fluids, contain RNase (a catalyst for RNA degradation) suggesting that viable miRNAs are somehow protected from RNase activity. Breast milk-derived miRNAs are believed to primarily originate from maternal mammary epithelial cells and immune cells but could potentially also originate from cells in other parts of the body, reaching the breast milk through the blood circulation (12, 25). However, it is assumed that the majority of human breast milk miRNAs are not found freely in breast milk but rather encapsuled in “vehicle” structures; primarily extracellular vesicles (EVs) (26). The EVs are bilayer membraned vesicles involved in intercellular signaling, and transport of proteins, nucleic acids and lipids from the originating cell to the target cell. Packing of EVs involves sorting mechanisms that favor some cargos over others and is hence not random (27, 28); emphasizing the biological relevance of their content.

*In vitro* studies have shown that milk-derived EVs survive when exposed to the harsh conditions of gastric digestion (29–31) and are even able to subsequently enter intestinal crypt-like cells (29–31). Furthermore, *in vivo* animal experiments show that exogenous miRNAs can be absorbed through the digestive tract only to be further distributed throughout the body of a suckling pup or piglet (32–34), or after oral gavage in adult mice (35). Cells seem to absorb EVs *via* different endocytic pathways, including clathrin-dependent endocytosis, phagocytosis, macropinocytosis and caveolin-mediated uptake, but the mechanisms in how the miRNA loaded EVs are absorbed by the epithelial cells in the infants’ intestine remain to be discovered in detail (13). Nevertheless, the fact that milk-derived miRNAs to a large extent are evolutionary conserved and can survive the harsh conditions of the digestive tract to be taken up in the intestine of the offspring, points to the important influence they may have on the epigenetic development of the child.

### 1.2 Immunological tolerance

One of the earliest biological challenges in life is the ability of the infant’s immune system to distinguish between harmful and harmless proteins, also known as antigens. An adverse immune response to food antigens is known as food allergy. The prevalence of food allergy is believed to be at an all-time high, although describing the increasing rates in exact numbers proves a challenge (36–39). Nevertheless, allergies pose a great challenge to the afflicted individual and their next of kin (40), and in the absence of a cure (41, 42) further mechanistic insight is warranted.

**Figure 1:**
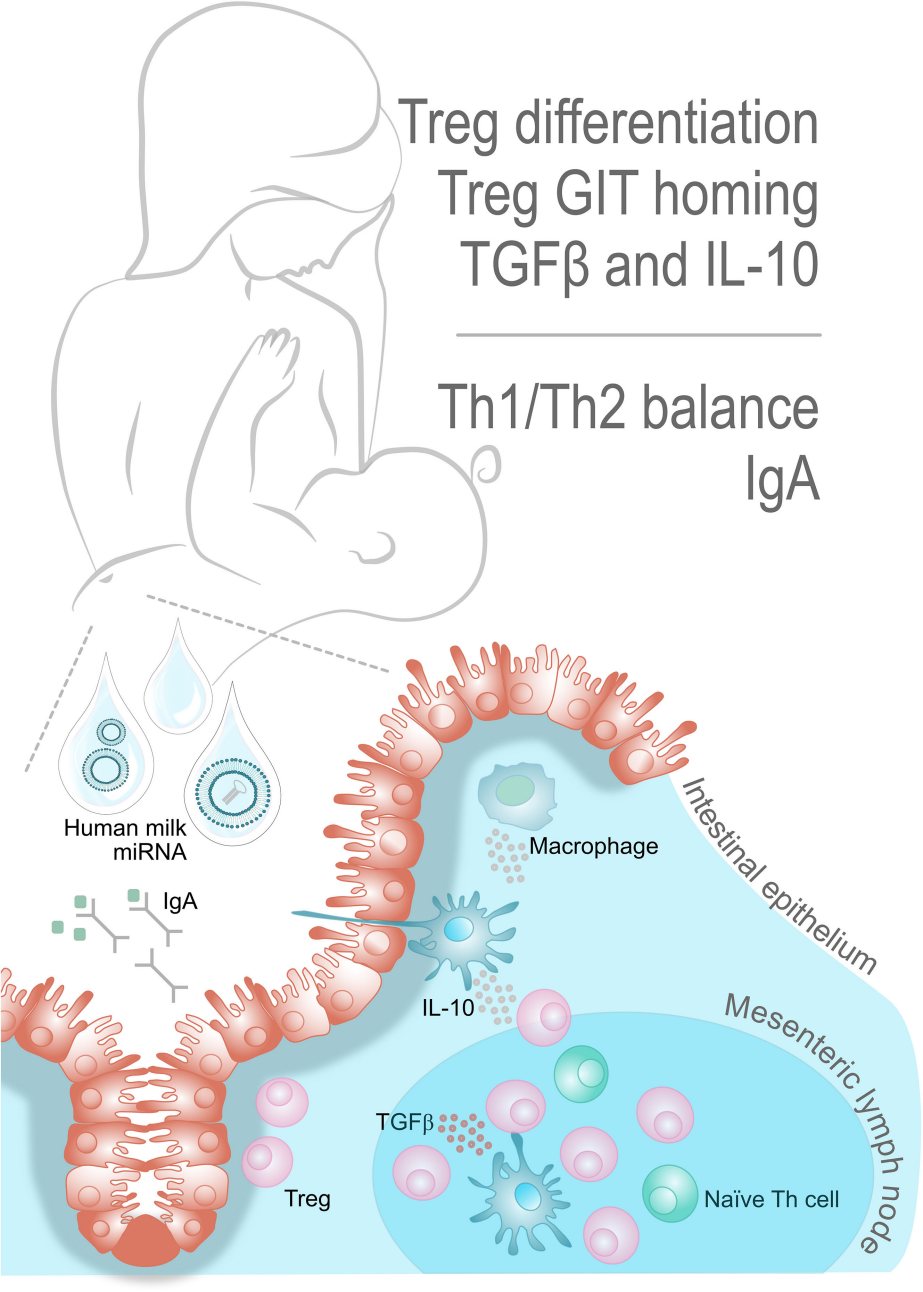
Key players in oral tolerance. Oral tolerance is an active suppression of cellular and humoral responses to antigens. The mesenteric lymph node is the major site for Treg cell differentiation, as mediated by CD103+ DCs. Production of the anti-inflammatory cytokines IL-10 and TGF-β supports the positive feedback loop of stimulating CD103+ DCs, Tregs and tolerogenic macrophages. The TGF-β also promotes B cell antibody class switching towards IgA, thus facilitating antigen exclusion in the intestinal lumen. Interestingly, there seems to exist several breastmilk miRNAs with potential involvement in these interactions.

The immune system avoids adverse reactions to foods through the induction of oral tolerance (43); see the key factors, as described below, depicted in **Figure 1**. Tolerance, i.e. systemic and mucosal unresponsiveness, is maintained through interaction between intestinal cells and immune cells at mucosal surfaces. In the gastrointestinal tract (GIT), where the infant is exposed to breast milk miRNAs, the gut-associated lymphoid tissues including the mesenteric lymph nodes (44) (MLN), play a major role in these interactions. The gut-associated lymphoid tissues include both innate and adaptive immune cells and tolerance is primarily developed through interaction between regulatory dendritic cells (CD103+ DCs) and naïve T helper (Th) cells in the MLN, where the CD103+ DCs promote Th cell differentiation into Tregs. The Tregs, will favor a tolerogenic immune response by producing the anti-inflammatory cytokines IL-10 and TGF-β, inhibiting the proliferation of effector T cells and thereby mitigating adverse inflammatory responses (45). Tregs are hence at the hub of oral tolerance development. The TGF-β produced by Tregs also promotes B cell antibody class switching towards immunoglobulin (Ig) A, facilitating IgA-mediated antigen exclusion in the intestinal lumen (46). Moreover, the CD103+DCs of the MLN also imprint GIT homing, so the Tregs may leave the lymph node and migrate back to the lamina propria. In fact, induction of Treg homing back to the gut has been suggested as a vital step to install oral tolerance (46, 47). In the gut, Tregs are exposed to IL-10 producing macrophages that cause their clonal expansion and thereby facilitate their suppressive function (43, 47). As pointed out above, more than one breast milk miRNA has been previously implicated in these interactions, hence, we hypothesize that breast milk miRNAs are important for installing immunological tolerance in the infant.

### 1.3 Aim

In this review we set out to predict the mRNA targets of the top 10 expressed miRNAs in human breast milk, focusing on their relevance in oral tolerance development and allergy prevention in the infant.

## 2 Methods

**Figure 2:**
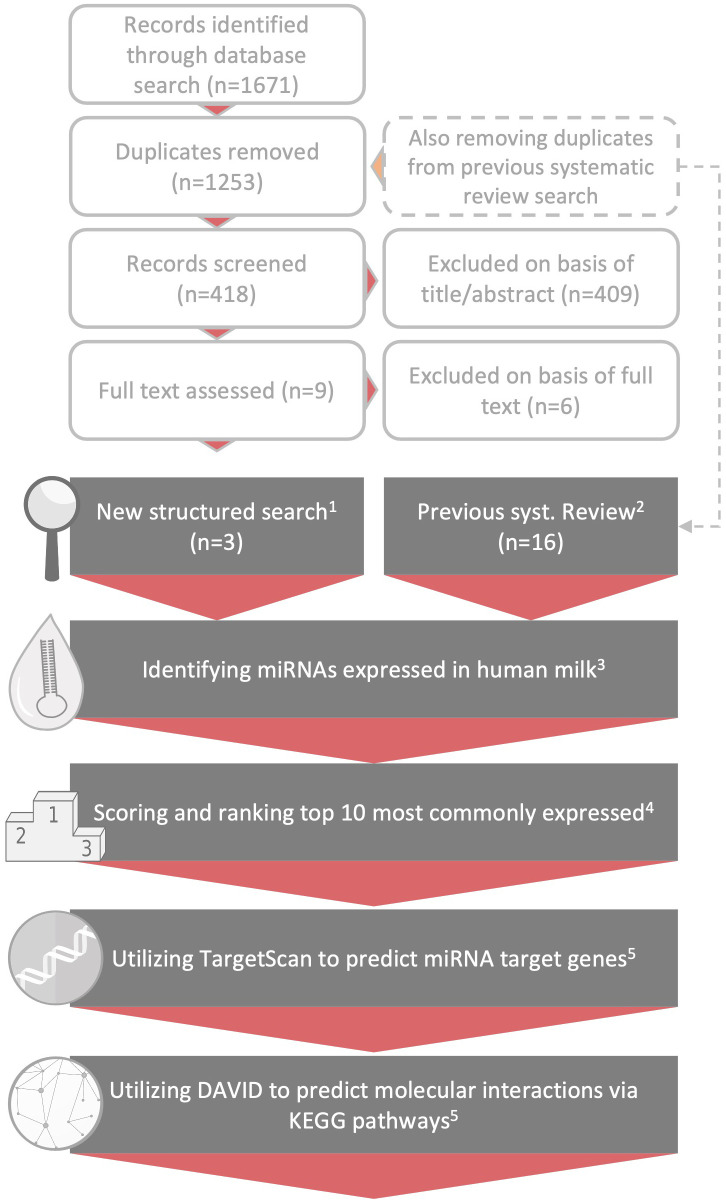
Methodological overview. Overview of the methodological process. The topmost light grey, unfilled text boxes depict the paper selection following the updated literature search. Below, depicted by the grey text boxes, follows the steps taken for the result generation: 1) and 2) illustrates how the three papers included after the new search was combined with the 16 papers analyzing *miRNA* in breast milk from our previous ncRNA review (14), 3) on basis of these 19 papers we identified the highest expressed breast milk miRNAs in each study (Supplementary B, **Table S1**), 4) thereafter the miRNAs were scored according to paragraph 2.2 (also refer to Supplementary B, **Tables S2-S5**) and for the last two steps denoted 5) please refer to method paragraph 2.2 and Supplementary B, **Tables S6-S8**.

For a visual overview of the methods, please refer to **Figure 2**.

### 2.1 Literature search

The top 10 expressed miRNAs in human breast milk were identified based on a previous systematic review produced by us in 2021 (14) (PROSPERO Identifier: CRD42020138989), where we summarized the scientific studies on non-coding RNAs (ncRNA) in breast milk up until September 2020. To also capture relevant literature published after this date, a new structured search was conducted in the bibliographic databases MEDLINE, Cochrane Library, Embase and Web of Science. The search strategy included thesaurus- and free-text terms for the two main concepts miRNA and mothers’ milk (see Supplementary A for complete details). The searches were last updated on the 9th of August 2022. The identified records were collected in EndNote and screened for duplicates (by author S.A.P). Records previously screened in the systematic review (14) were also removed. Following duplicate removal, the new records were screened for inclusion based on titles and abstract by author E.A. and subsequently cross-checked by author M.R.S.

Please observe that in updating our previous search, we included new papers within the area of miRNA and mothers’ milk research, as opposed to the previous systematic search where we considered all ncRNAs. Hence, although this paper in some respects draws from our previous systematic review, it should be considered as a stand-alone paper built on a separate rational and aim.

### 2.2 Ranking of the miRNAs, target prediction and pathway analysis

Functional analysis was performed on the top 10 expressed miRNAs in breast milk based on the previous summary in Tingö et al. (14), including 16 papers (15, 23, 25, 29–31, 48–57) and adding information from 3 additional papers found in the updated literature search described above (17, 58, 59). The most frequently expressed miRNAs, as described either by the authors or calculated based on the data from their corresponding supplementary information, were based on RNA sequencing or open Array qPCR analysis and isolated from all fractions, i.e. whole, cell, skim milk, lipid or EVs (refer to Supplementary B, **Table 1**). From each included study, the top 10 expressed miRNAs were assigned a score between 1 and 10 that corresponded to their expression ranking (*i.e*., the highest expressed miRNA was assigned a score of 10; see complete scoring in Supplementary B, **Table S1**), to get an average of the top 10 most abundant miRNAs from the 19 included papers. The miRNAs belonging to the same family were scored together (Supplementary B, **Table S2**) due to their shared seed region and thus common mRNA targets. The assigned scores for each miRNA were then summarized across studies, producing a total score for each miRNA on basis of their ranking (Supplementary B, **Table S3**); miR-6073 was removed due to the likelihood that the high relative abundance of this miRNA in two studies may be an artifact of the adopted sequencing protocol as discussed in our previous review (14). Furthermore, the miRNA-200 family consists of miR-200a/b/c, miR-141 and miR-429, however, they are divided into two groups based on their seed region similarities; miR-200a and miR-141-3p in one group, and miR-200b/c and miR-429 in the other (60). The groups were scored separately, and subsequently included among the top 10 expressed (Supplementary B, **Table S3**).

**Table 1:** The top 10 highest ranked human breast milk miRNAs.

Top 10 miRNA families	Top 10 individual miRNAs
let-7-5p family	miR-148a-3p
miR-30-5p family	miR-22-3p
miR-148a-3p	miR-30d-5p
miR-200a-3p + miR-141-3p^a^	miR-30a-5p
miR-22-3p	miR-146b-5p
miR-181-5p family	miR-141-3p
miR-146b-5p	let-7a-5p
miR-378a-3p	miR-181a-5p
miR-29-3p family	let-7f-5p
miR-200b/c-3p^a^	let-7b-5p

The miRNAs are presented in falling order, i.e. the miRNAs at the top of the lists were found to be the most highly expressed after taking into account the results from all 19 studies included in the review. The miRNA presented as families in the left column were scored together as they share the same seed region. In the right column we list the individually top expressed miRNAs, i.e. the ranking after all miRNAs were scored as singlets. a) The miRNA-200 family consists of miR-200a/b/c, miR-141 and miR-429, however, they are divided into two groups based on their seed region similarities; miR-200a and miR-141-3p in one group, and miR-200b/c and miR-429 in the other. Hence, the two groups were scored separately. The individual miRs shared by both lists are bolded.

**Figure 3:**
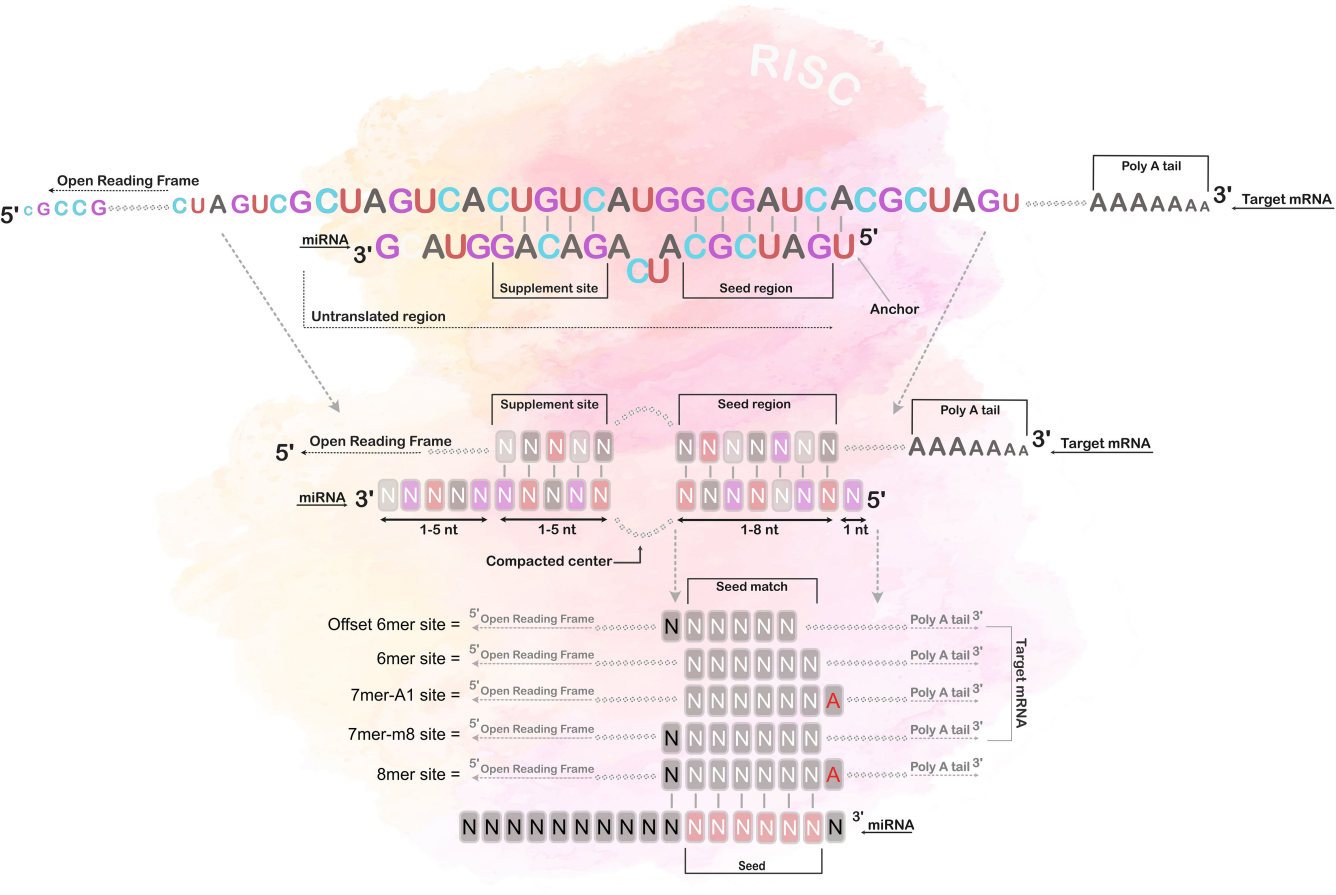
Seed region matching. The figure illustrates the different types of canonical site paring between the miRNA and its mRNA target, *i.e.* 8mer, 7mer-1A, 7mer-m8 and 6mer paring. The 8mer paring has a complete matched seed region of 7 bases, including an additional match at the 8th position, and an adenine opposite of the first position; the 7mer-1A has a match between position 2-7 and an adenine opposite of the first position; the 7mer-m8 has a match between position 2-8, and the 6mer has a match only in position 2-7. Notably, most miRNAs also have additional pairing beyond the seed region, called 3’-supplementary site.

Target prediction was run using TargetScan version 8.0 (61, 62), with the default settings, and an upper threshold for the cumulative weighted context++ score was set at -0.2 (62). The prediction of targets are primarily based on the mRNA matching at the “canonical” site, i.e. a base sequence matching perfectly to the miRNA seed region (62, 63), and a set of additional variables (e.g., sequence conservation, target site accessibility, flanking sequence determinants and compensatory paring outside the seed region) that contribute to reducing the number of false positive predictions (61, 64), refer to **Figure 3** for visual illustration. However, contrary to many other prediction algorithms, TargetScan also base the predictions on an additional set of variables, such as sequence conservation, target site accessibility, flanking sequence determinants, and compensatory pairing outside the seed region, which reduce the number of false positive predictions (61, 64).

The lists of predicted target genes were subsequently uploaded to the Database for Annotation, Visualization and Integrated Discovery (DAVID) 2021 (65, 66), and potential biological functions of the top 10 miRNAs were analyzed using the Kyoto Encyclopedia of Genes and Genomes (KEGG) pathway enrichment analysis. An FDR corrected p-value < 0.05 was considered of interest.

## 3 Results

### 3.1 Literature search

A total of 1671 records were identified by the updated literature search. After removal of duplicates, and records previously screened in our systematic review on ncRNAs in human breast milk (14), 418 unique and new records were identified and subjected to further screening. Out of these 418 records, 5 studies were read in full text and 3 were subsequently judged as relevant for this review. The results of these 3 studies were subsequently combined with the results of the other 16 studies originating from the systematic review, producing a grand total of 19 scientific studies (based on 25 unique data sets) serving as the basis of this review.

### 3.2 Top 10 expressed miRNAs in human milk

The top expressed miRNAs, from the 19 included studies, were scored and ranked; for ranking see **Table 1**, and for further details in the scoring system refer to the method section and Supplementary B, **Tables S1-S5**). Accordingly, the list of the most highly expressed breastmilk miRNAs in falling order is as follows: let-7-5p family, miR-30-5p family, miR-148a-3p, miR-200a-3p + miR-141-3p, miR-22-3p, miR-181-5p family, miR-146b-5p, miR-378a-3p, miR-29-3p family and lastly miR-200b/c-3p.

Note that we consider the familywise ranking of the miRNAs to be our main result (see **Table 1**, left column). The rationale behind this is that miRNAs sharing seed region will interact with the same mRNA targets and are thus likely to produce a more pronounced physiological effect collectively, as compared to a single miRNA. **Table 1**, however, also shows the top expressed miRNAs ranked individually as this may be interesting complementary information.

### 3.3 Target prediction and pathway analysis

In the target prediction search we identified 3,588 potential target genes using TargetScan 8 (see complete list in Supplementary B, **Table S6**). The predicted genes resulted in 127 KEGG pathways of interest based on FDR corrected p-values < 0.05. Several of the pathways were directly connected to the immune system, including TGF-beta signaling, T cell receptor signaling, Toll-like receptor signaling, Jak-STAT signaling, and Th1 and Th2 cell differentiation (see complete list in Supplementary B, **Table S7**). In addition, some major signaling pathways also connected to immune regulation, such as the PI3K-Akt signaling pathway, MAPK signaling pathway and, FoxO signaling pathway were also indicated.

## 4 Discussion

This review set out to predict the mRNA targets of the top 10 expressed miRNAs in human breast milk, focusing on their possible role in infant immunological maturation and more specifically on oral tolerance development and allergy prevention. In combining the findings from a previous systematic review from our research group (14) with an updated database search, we found the following miRNAs to be the most highly expressed in human milk: let-7-5p family, miR-148a-3p, miR-30-5p family, miR-200a-3p+ miR-141-3p, miR-22-3p, miR-181-5p family, miR-146b-5p, miR-378a-3p, miR-29-3p family, miR-200b/c-3p and miR-429-3p. Together these miRNAs were predicated to interfere with 3,588 gene products and when weighed together for pathway prediction these targets were implicated in 127 KEGG pathway. Several of these pathways were directly connected to the immune system, including TGF-beta signaling, T cell receptor signaling, Toll-like receptor signaling, Jak-STAT signaling, and Th1 and Th2 cell differentiation. In addition, some major signaling pathways frequently indicated in normal physiology, but also highly relevant for immune regulation, such as the PI3K-Akt signaling, MAPK signaling, TNF signaling and FoxO signaling, were also indicated.

Here, we have used a systematic strategy to find original studies analyzing breast milk miRNA and subsequently developed a system to identify out the most commonly expressed ones. To narrow down the number of miRNAs to be addressed in this review, we chose to focus on the top 10 miRNAs from the included studies. Of course, this potentially means disregarding miRNAs that have important immunological effects albeit expressed in low amounts. For example, miR-155 did not make it onto our list. This miRNA has received rather much attention due to its presumed involvement in Treg differentiation; it has been previously demonstrated that miR‐155 promotes the differentiation of Treg in allergic rhinitis (18). Similarly, Hicks et al. (16) recently showed that breast milk miR-375 seem to decrease the risk of atopy in breastfed infants; a miRNA that did not make it on to our top 10 list. Nevertheless, while acknowledging this as a potential shortcoming, we believe that adhering to a systematic approach in selecting which miRNAs to focus on adds rigor to this review. Furthermore, for additional information on methodological differences between the studies, such as sample source, milk fraction and maternal characteristics, please refer to our previous review (14), where we systematically deal with these topics. Moreover, we choose to focus on the pathways and corresponding targets of the top 10 expressed miRNAs from an oral tolerance perspective, as we hypothesize that breast milk miRNAs are important in immunological maturation and childhood allergy prevention. In this context, it is important to note that this review, albeit based on a systematic approach, is speculative in nature. It is also important to note that we do not attempt to predict the direction of action, *i.e.* suppressive or stimulatory effects. In general, miRNA act inhibitory as their primary function, but in instances where a miRNA ends up suppressing a suppressor the effect will turn out stimulatory. As the databases utilized here predict direct seed matching and enrichment of targets in the indicated pathways, making predictions about such indirect suppressive actions are difficult. The inhibitory action of a miRNA is likely also a delicate balance between the potency of the miRNA and the expression of the target mRNA at different levels. Additionally, each miRNA has several targets that are also shared with other miRNAs. Due to the level of complexity of these interactions, we deemed direction of action to be outside the scope of the current review; settling on the mere fact that human milk is enriched with miRNAs of significance to these pathways/interactions gives reason to speculate about their importance in oral tolerance development. Furthermore, we make the underlying assumption that breast milk miRNAs are protected from degradation, most likely by EV encapsulation, and taken up in sufficient amounts to exert biological effects in the infant recipient cells. As outlined in the introduction, this notion has some previous support in the scientific literature as milk miRNAs are taken up by suckling animals. However, the route of uptake is not yet completely clarified. Endocytosis is most likely one prominent way of recipient cells to incorporate exosomal miRNAs (67, 68), or less supervised uptake may potentially occur in immune cells such as macrophages (69, 70). In infants, the immature state of the intestine may further facilitate uptake of EV and their miRNA cargo, as the neonatal GIT show increased permeability (as reviewed in (71)).

As further laid out in the introduction of this paper, the primary site for oral tolerance induction is the GIT, where the infant is also exposed to breast milk. In the MLN, CD103+ DCs interacts with naïve Th cells to induce Treg differentiation; this process is highly TGF-β dependent. Among the top expressed breast milk miRNAs, we find direct mRNA targets upstream of TGF-β (miR-148a-3p) and the TGF-β receptors 1/2/3 (miR-181-5p and let-7-5p). In addition, miR-148a-3p have targets within the SMAD-family; activation of TGF-β receptor leads to the activation of the Smad downstream signaling cascade, promoting FoxP3 expression (72). The chemokine receptor CCR7 is involved in the egress of Tregs from tissues to lymph nodes (73–76) and seem to be crucial for Treg induction by antigen presenting cells primed by commensal microbes (77); CCR7 is a direct target of the breast milk expressed let-7-5p. The clonal expansion of the Tregs is further promoted by IL-10 producing macrophages; the IL-10 mRNA is a direct target of let-7-5p, while miR-29-3p targets the IL-10 receptor beta. A recent study, conducted in humans and mice, demonstrated that miR‐181a regulates the expression of IL‐10 and TGF‐β in allergic rhinitis (18). In addition, IL-10 and TGF-β are also important factors in facilitating Treg inhibition of effector T cell proliferation. Importantly, previous research has shown that deficiency in TGF-β or its receptors is proven to be fatal in the first few weeks of life, due to fulminant inflammation (78). Interestingly, TGF-β deficient mouse pups remains healthy as long as they are fed milk from dams that are not TGF-β deficient, but develop severe inflammation when weaned (79). As such TGF-β is crucial for stimulation of naïve CD4+ T cells to differentiate into Foxp3+ Treg and their subsequent suppression of effector T cell activation and proliferation, as this will halt the life-threatening inflammatory response. It is noteworthy that DICER (an enzyme crucial for miRNA biogenesis) knock out mice have low number of Tregs and develops fatal autoimmune conditions (80–82).

The combination of TGF-β with other cytokines could push naïve T cells to differentiate into non-regulatory T cell subtypes, for example Th17 differentiation is promoted in the presence of IL-6 (72, 83). Th17 cells are pro-inflammatory in their action and in many instances are regarded as problematic due to their involvement in autoimmunity and related tissue damage (84, 85). Failure of oral immune therapy, aimed to treat peanut allergy, was associated with the expression of inflammatory gene signatures present in Th17 cell populations (86), also indicating a role for Th17 cells in oral immunotherapy efficacy. The mRNA of the Th17 key transcription factor RORC is a let-7-5p target, as is the IL17 receptor IL17RD. The RORC transcription factor (known as RORγt in mice), however, also seems important for a suppressive subset of Foxp3 expressing RORγt+ T cells with regulatory function against exacerbated Th2 responses and linked to oral tolerance development in mice (87). These RORγt+ Tregs can be generated under the influence of IL-2 and TGF-β, and despite their Th-17 shared characteristics they seem to have suppressive activity against antigen-specific effector T cells *in vitro* (88). In addition to let-7 targeting RORC and IL-17, miR-30-5p have a target in the IL-2-receptor a mRNA. The IL-17 signaling pathway was also indicated in the KEGG pathways analysis. Interestingly, these double positive Foxp3+RORγt+ T cells have been found in the small intestinal and colonic lamina propria of mice, linked to a specific, but diverse set of bacterial species (89–91). In addition, colonization of germ-free mice with Clostridia species upregulates IL-22 production by RORγt+ innate lymphoid cells and T cells in the lamina propria of the intestine, contributing to a reduced permeability to oral antigens (92). This is interesting as tight junctions between enterocytes in the GIT prevent the paracellular passing of antigens. An increased transport of intact antigens through the epithelial cells has been related to allergenic activity (93, 94). On this note, we also found several targets involved in tight junction regulation, e.g. miR-200b-c-3p targets the occludin mRNA.

Inoculation of the infant gut with the maternal microbiota during and after birth is thought to promote an accelerated transition from Th2 to a Th1/Th17-dominant immunity, as well as facilitating Treg induction in the gut and lungs (95). A Th2 skewed immune system is the prevailing antenatal programming, meant to accommodate fetal development and promoting maternal tolerance towards the fetus (96). Th2-mediated immune responses are, however, also associated with allergic reactions mediated by B-cell produced IgE antibodies. A Th1-mediated response on the other hand antagonizes IgE responses and hence, hypothetically, prevents the development of allergic disease. Therefore, a lingering post-natal Th2 programming may increase the risk of allergy development in the infant. Indeed, previous research from our group has shown that placental gene expression, including cells of fetal origin, is dominated by a Th2- and anti-inflammatory transcription profile and that enhanced Th2 deviation at birth is related to increased risk of allergy development in the child (97, 98). Tregs are key players in the calibration of Th2 as well as Th1/Th17 driven inflammatory responses and are thus crucial to avoid adverse immune reactions (99–101). The gut microbiota also seems to play a key role in oral tolerance induction, for example by regulating the phenotype of GIT DCs (mediated by macrophages and innate lymphoid cells) (102) and Tregs (103, 104). Babies delivered by Cesarean section have a different microbiota composition early in life as compared to vaginally delivered infants (105–107) and the former are also at greater risk of developing allergies later in life (106, 108). Interestingly, the disturbance of gut microbiota in cesarean delivered infants may to some extent be restored by breastfeeding (109, 110).

The toll-like receptor (TLR) signaling pathway was indicated among the KEGG pathways. The TLRs are pattern recognition receptors that respond to microbial signals, and in particular TLR2 has been implicated in oral tolerance by promoting Treg induction as mediated by DCs (111). Soluble TLR2, capable of modulating TLR2 signaling, has also been found in human milk (112). A previous study by our group suggests that probiotic supplementation in children may decrease responses to TLR2, potentially dependent on factors downstream of TLR mRNA expression (113); which hence hypothetically could be miRNA mediated. The probiotic intervention in our study also reduced allergen responsiveness (114), and IgE-associated eczema at two years of age (115). As such, TLRs may be regarded as an important “bridge” between gut microbiota and tolerance development. In addition, the C-type lectin receptor signaling pathway was also indicated among the breast milk miRNA predicted KEGG pathways; the C-type lectin domain family 2 is a target of breast milk miRNA miR-29-3p. As the TLRs, the C-type lectin receptors (CLRs) represent a family of PPRs, recognizing both pathogens associated molecular patterns and damage-associated molecular patterns. Upon activation, CLR signaling leads to cytokine secretion and immune cell recruitment, which portraits their role in inducing innate and adaptive immune responses (116). The dendritic cell-associated C-type lectin-1 (Dectin-1) is one of the most well studied CRLs, which has been implicated in bridging the innate and adaptive immune responses. For example, dectin-1 activates the transcription factor NF-κB, through both canonical and noncanonical pathways, initiating its signaling pathway to induce Th1 and Th17 cytokine production (117), and dectin-1-Syk-CARD9 signaling seems to promote DC maturation and Th17 differentiation, both *in vitro* and *in vivo* (117).

IgE-mediated allergy reactions are caused by B-cell produced IgE antibodies triggering mast cell activation through cross-linking with the high affinity Fc epsilon Receptor I (FcϵRI) on the mast-cell surface (118, 119). Notably, miR-22 together with miRNAs from the let-7-5p family have direct targets in the IL-13 mRNA and its alpha 1 receptor; IL-13 is a central regulator in IgE synthesis and has been described as an important mediator of allergic inflammation (120). In addition, we found predicted targets in FcϵRI signaling and the B-cell receptor signaling pathway. The FcϵRI exists on mast cells and basophils. Upon cross-linking with IgE, the FcϵRI induces the release of pre-made histamines through mast cell degranulation and triggers the synthesis of a wide range of pro-inflammatory cytokines and chemokines, alongside leukotrienes and prostaglandins (118, 119, 121). Similarly, IgE-mediated activation of basophils will lead to the release of various proinflammatory mediators, but especially IL-4 and IL-6 (122). Cytokine signaling is mediated through the Janus kinase-signal transducer and activator of transcription (JAK-STAT) pathway, which was also indicated among our predicted KEGG pathways. The JAK-STAT signaling cascade is responsible for transferring signals from cytokine receptors to the nucleus, making the JAK-STAT pathway heavily involved in regulation of the immune system (123). Other than the cytokines, receptors and chemokines already mentioned in this review we found that both IL-1A and IL-31, as well as IL-1RAP, IL-1RAPL2, IL-2RA, and CCL3/7/8 are targets among the top expressed miRNAs found in breastmilk.

Furthermore, concerning the connection between allergy development and balance in Th1 versus Th2 responses, miR-29 directly targets the TBX21 mRNA (also known as T-bet), the key transcription factor of Th1 cells (124), and miR-181-5p targets CD4, a co-receptor of the T cell receptor expressed on Th cells. Indeed, previous research from our group have shown that enhanced Th2 deviation at birth is related to allergy development in the child (97, 98). Furthermore, we have also shown that breast milk has anti-inflammatory effects on peripheral blood mononuclear cells from cord blood (125); the high levels of TGF-β in breast milk and inhibitory effect of colostrum on IL-4 production, suggests a possible mechanism whereby breast milk of certain composition may protect against the development of allergy. We emphasize here that “certain compositions” may be of particular interest since previous studies on the preventative effects of breast milk on allergy are inconclusive (126–130). However, there remains a possibility that varying levels of breast milk mediators may partially explain the previous conflicting findings (131). We have for example shown that the concentrations of the Th2 associated cytokines IL-4, IL-5 and IL-13 seem to be higher in breast milk from allergic compared to non-allergic mothers (132) and that breast milk cytokine levels may have geographical variations, perhaps depending on differences in microbial load (133). Indeed, members from our group recently showed that allergy development is associated with consumption of breastmilk with a reduced microbial richness in the first month of life (134). There are also indications that levels of certain breast milk miRNA differ between allergic and non-allergic women, for example, downregulation of let-7f-5p in mature milk tends to be associated with development of atopic dermatitis in breastfed infants (50). Hence, the composition likely matters; not all breastmilk is created equal. Whilst composition likely matters, the scientific literature investigating these relations is still fairly scarce, especially in terms of mechanisms and causality, and future research will have to bring further clarity to these interactions.

### 4.1 Future directions

In this review we make a case for breastmilk miRNAs as important regulators of infant immune maturation, focusing on their potential role in oral tolerance development and allergy prevention. This review is speculative in nature and future studies are needed to confirm the ideas put forward here, and particularly to bring further clarity to the direction of action in the interactions suggested. This will require well-designed trials in an *in vivo* setting, but also functional experiments uncovering the underlying mechanisms of action *in vitro*. We would also like to point out that the 19 scientific studies serving as the basis of this review were all performed in women from high-income countries; no studies have yet been performed outside of this population segment.

### 4.2 Conclusion

Infant immune regulation is a complex process impacted by multiple factors and relies on the finetuning of multiple factors related to Th differentiation. For example, DCs and Th interactions in the MLN, and Treg GIT homing play a central role in tolerance development as the primary site of interactions between food antigens, immune cells, gut microbiota, and the immunologically active components in breast milk. Here, we highlight the role of breast milk miRNAs and their potential contribution to infant immune maturation. Indeed, breast milk miRNAs seem to be involved in several pathways that may have implications for oral tolerance development. However, clarification of the direction of action in these complex interactions will require knowledge production from cleverly designed functional experimental and further studies of the co-variation of these factors in *in vivo* systems.

## Author contributions

LT, EA, AA-K and RT designed research. SP performed the updated database search. EA and MS reviewed results from the literature search. EA and MS compiled the list of the top 10 expressed miRNAs. EA performed target and pathways predictions. LT, EA, AA-K and RT wrote the paper. LT did all figures. MJ, EC, SP, MS edited the paper. LT had primary responsibility for final content. All authors contributed to the article and approved the submitted version.
