# Supplementary material for: Breast milk microRNAs: Potential players in oral tolerance development
Source: Front Immunol. 2023 Mar 14;14:1154211. doi: 10.3389/fimmu.2023.1154211 (PMC10045994; doi:10.3389/fimmu.2023.1154211)
Supplement: Supplementary file 1 [file DataSheet_1.docx]

Supplementary Material

**Breast milk microRNAs: potential players in oral tolerance development**

**Emelie Ahlberg^1^**†**, Ahmed Al-Kaabawi^1^**†**, Rebecka Thune^1^, Melanie Rae Simpson^2^, Sindre Andre Pedersen^3^, Erika Cione^4^, Maria C. Jenmalm^1^, Lina Tingö^1,5,6^***

† These authors share first authorship

*** Correspondence:**Lina Tingö, lina.tingo@liu.se

# Supplementary A

## MEDLINE

Ovid MEDLINE(R) ALL <1946 to August 05, 2022>

| 1. | exp Colostrum/ |  |  |  |  |  |
| --- | --- | --- | --- | --- | --- | --- |
| 2. | exp Breast Feeding/ |  |  |  |  |  |
| 3. | exp Lactation/ |  |  |  |  |  |
| 4. | exp Milk/ |  |  |  |  |  |
| 5. | (breastmilk or breast-milk or mothersmilk or mothers-milk or ((breast or human? or maternal or mother? or woman) adj2 milk)).ti,ab,kf. |  |  |  |  |  |
| 6. | (breastfe* or breast-fe* or (breast adj2 (feed* or fed))).ti,ab,kf. |  |  |  |  |  |
| 7. | (colostrum* or colostral* or colostrium or foremilk or fore-milk or hindmilk or hind-milk).ti,ab,kf. |  |  |  |  |  |
| 8. | (lactation or breast-secretion* or milk-release or ((breast or milk) adj2 (secretion* or release))).ti,ab,kf. |  |  |  |  |  |
| 9. | or/1-8 [Concept #1: Breast milk] |  |  |  |  |  |
| 10. | MicroRNAs/ |  |  |  |  |  |
| 11. | Circulating MicroRNA/ |  |  |  |  |  |
| 12. | micro-ribonucleic-acid?.ti,ab,kf. |  |  |  |  |  |
| 13. | microRNA?.ti,ab,kf. |  |  |  |  |  |
| 14. | micro-RNA?.ti,ab,kf. |  |  |  |  |  |
| 15. | miRNA?.ti,ab,kf. |  |  |  |  |  |
| 16. | mi-RNA?.ti,ab,kf. |  |  |  |  |  |
| 17. | miRs.ti,ab,kf. |  |  |  |  |  |
| 18. | or/10-17 [Concept #2: miRNA] |  |  |  |  |  |
| 19. | and/9,18 [Concept #1 and #2 combined] |  |  |  |  |  |

**1.2 Cochrane Library**

1. MeSH descriptor: [Colostrum] explode all trees

2. MeSH descriptor: [Breast Feeding] explode all trees

3. MeSH descriptor: [Lactates] explode all trees

4. MeSH descriptor: [Milk] explode all trees

5. ((breastmilk or breast-milk or mothersmilk or mothers-milk or

((breast or human? or maternal or mother? or woman)

NEAR/2 milk))):ti,ab,kw (Word variations have been searched)

6. ((breastfe* or breast-fe* or (breast NEAR/2 (feed* or fed))))

:ti,ab,kw (Word variations have been searched)

7. ((colostrum* or colostral* or colostrium or foremilk or fore-milk

or hindmilk or hind-milk)):ti,ab,kw (Word variations have been

searched)

8. ((lactation or breast-secretion* or milk-release or ((breast or milk)

adj2 (secretion* or release)))):ti,ab,kw (Word variations have been

searched)

9. #1 OR #2 OR #3 OR #4 OR #5 OR #6 OR #7 OR #8

10. MeSH descriptor: [MicroRNAs] explode all trees 250

11. MeSH descriptor: [Circulating MicroRNA] explode all trees

12. (microRNA?):ti,ab,kw (Word variations have been searched)

13. (micro-RNA?):ti,ab,kw (Word variations have been searched)

14. (miRNA?):ti,ab,kw (Word variations have been searched)

15. (mi-RNA?):ti,ab,kw (Word variations have been searched)

16. (miRs):ti,ab,kw (Word variations have been searched)

17. #10 OR #11 OR #12 OR #13 OR #14 OR #15 OR #16

18. #9 AND #17 45

**1.3 Embase**

Embase <1974 to 2022 August 05>

| 1. | exp Colostrum/ |  |  |  |  |  |
| --- | --- | --- | --- | --- | --- | --- |
| 2. | exp Breast Feeding/ |  |  |  |  |  |
| 3. | exp Lactation/ |  |  |  |  |  |
| 4. | Breast milk/ |  |  |  |  |  |
| 5. | (breastmilk or breast-milk or mothersmilk or mothers-milk or ((breast or human? or maternal or mother? or woman) adj2 milk)).ti,ab,kw. |  |  |  |  |  |
| 6. | (breastfe* or breast-fe* or (breast adj2 (feed* or fed))).ti,ab,kw. |  |  |  |  |  |
| 7. | (colostrum* or colostral* or colostrium or foremilk or fore-milk or hindmilk or hind-milk).ti,ab,kw. |  |  |  |  |  |
| 8. | (lactation or breast-secretion* or milk-release or ((breast or milk) adj2 (secretion* or release))).ti,ab,kw. |  |  |  |  |  |
| 9. | or/1-8 [Concept #1: Breast milk] |  |  |  |  |  |
| 10. | micro-ribonucleic-acid?.ti,ab,kw. |  |  |  |  |  |
| 11. | microRNA?.ti,ab,kw. |  |  |  |  |  |
| 12. | micro-RNA?.ti,ab,kw. |  |  |  |  |  |
| 13. | miRNA?.ti,ab,kw. |  |  |  |  |  |
| 14. | mi-RNA?.ti,ab,kw. |  |  |  |  |  |
| 15. | miRs.ti,ab,kw. |  |  |  |  |  |
| 16. | or/10-15 [Concept #2: miRNA] |  |  |  |  |  |
| 17. | and/9,16 [Concept #1 and #2 combined] |  |  |  |  |  |
|  | | |  |  | | |

**1.4 Web of Science**

1. ("breastmilk" or "breast-milk" or "mothersmilk" or "mothers-milk") (Topic)
Web of Science Core Collection

2. (("breast" or "human?" or "maternal" or "mother?" or "woman") NEAR/2 "milk") (Topic)
Web of Science Core Collection

3. ("breastfe*" or "breast-fe*") (Topic)

3. ("breast" NEAR/2 ("feed*" or "fed") ) (Topic)

4. ("colostrum*" or "colostral*" or "colostrium" or "foremilk" or "fore-milk" or "hindmilk" or "hind-milk") (Topic)

5. ("lactation" or "breast-secretion*" or "milk-release") (Topic)

6. (("breast" or "milk") NEAR/2 ("secretion*" or "release") ) (Topic)

7. #7 OR #6 OR #5 OR #4 OR #3 OR #2 OR #1

8. "micro-ribonucleic-acid?" (Topic)

9. "microRNA?" (Topic)

10. "micro-RNA?" (Topic)

11. "miRNA" (Topic)

13. "mi-RNA?" (Topic)

14. "miRs" (Topic)

15. #14 OR #13 OR #12 OR #11 OR #10 OR #9

16. #8 AND #15
